# Dietary Eggshell Membrane Powder Improves Survival Rate and Ameliorates Gut Dysbiosis in Interleukin-10 Knockout Mice

**DOI:** 10.3389/fnut.2022.895665

**Published:** 2022-05-19

**Authors:** Yongshou Yang, Huijuan Jia, Weida Lyu, Kyohei Furukawa, Xuguang Li, Yukio Hasebe, Hisanori Kato

**Affiliations:** ^1^School of Life Sciences, Anhui University, Hefei, China; ^2^Key Laboratory of Human Microenvironment and Precision Medicine of Anhui Higher Education Institutes, Anhui University, Hefei, China; ^3^Health Nutrition, Graduate School of Agricultural and Life Sciences, The University of Tokyo, Tokyo, Japan; ^4^ALMADO Inc., Tokyo, Japan

**Keywords:** inflammatory bowel disease, eggshell membrane, gut microbiota, IL10, colitis

## Abstract

Inflammatory bowel disease (IBD) is known to be associated with compositional and metabolic changes in the gut microbiota. The aim of this study was to investigate whether dietary eggshell membrane (ESM) improves survival rate or ameliorates gut dysbiosis in a spontaneous IBD model of interleukin-10 knockout (IL10^−/−^) mice. Female C57BL/6J wild-type (WT) and IL10^−/−^ mice (KO) were fed an AIN-93G basal diet or an ESM diet (KOE) for 19 weeks. Gut microbiota profiles were analyzed *via* 16S rRNA sequencing, and short-chain fatty acids in cecal content were analyzed with high-performance liquid chromatography. The results demonstrated that ESM supplementation significantly improved the survival rate and body composition in KO mice. Alpha diversity analysis of the microbiota revealed that ESM supplementation significantly increased gut microbial diversity, which was decreased in IL10^−/−^ mice. The Firmicutes/Bacteroidetes ratio was recovered to a normal level by ESM supplementation, suggesting that ESM helps maintain the compositional balance of the gut microbiota. ESM increased relative abundance of commensal bacterial Ruminococcus and Bacteroidales S24-7 and reduced the abundance of the proinflammatory-related bacterium, Enterobacteriaceae. Additionally, ESM supplementation promoted the production of butyrate in cecal contents and downregulated the expression of proinflammatory genes, including interleukin-1β (*Il-1*β) and tumor necrosis factor-α (*Tnf-*α) in IL10^−/−^ mice colon, indicating anti-inflammatory functions. These findings suggest that ESM may be used as a beneficial dietary intervention for IBD.

## Introduction

Inflammatory bowel disease (IBD) is characterized as a cluster of chronic gut inflammation disorders, including Crohn's disease and ulcerative colitis (1). Despite extensive efforts, the exact underlying mechanisms of IBD have not been clearly elucidated. However, several risk factors, including genetics, immune system, and environmental factors, play important roles in the development of IBD (2). Recently, abnormal microbial colonization of the gastrointestinal tract has been considered a possible pathogenesis of IBD in genetically susceptible individuals (3). Many previous studies have identified a perturbation of gut microbiome in both IBD patients and mouse colitis models (4–6). Compared to chemical-induced colitis models, interleukin-10 knockout (IL-10^−/−^) mice are often applied in mechanistic studies to examine the pathogeny of spontaneous, immune-mediated, chronic gastrointestinal inflammation (7, 8). Germ-free IL-10^−/−^ mice showed no proof of colitis or related immune system activation, suggesting the critical role of gut microbiota in IBD pathogenesis (9).

Current treatments for IBD mainly rely on anti-inflammatory drugs and surgery to relieve symptomatic complications, which have several side effects, including loss of immunotolerance and drug resistance (10). Administration of prebiotics, probiotics, or synbiotics has been demonstrated to ameliorate some colitis symptoms in both IBD patients and rodent colitis models (7, 11–13). These studies imply that targeting the gut microbiota is an effective strategy for IBD treatment (14).

Eggshell membrane (ESM) is a by-product of the egg process industry and is considered an environmental-burden waste. Our previous study found that dietary ESM exerts biochemical functions that can counter liver injury and fibrosis through modulating the PPARγ-endothelin 1 interaction signaling pathway (15). Moreover, dietary ESM was shown to ameliorate dextran sulfate sodium (DSS)-induced colitis through exerting anti-inflammatory effects and modulating the gut microbiota (16). The DSS-colitis model is widely used to mimic human IBD, mainly because of the massive epithelial injury and wholesale invasion of the lamina propria by gut microbiota, however, this is unlikely to be a crucial mechanism of human IBD (17). IL-10^−/−^ mice display chronic intestinal inflammation through impaired immunoregulatory function of antigen-presenting cells as well as dysfunction of macrophages (17). The efficacy of ESM in IL-10^−/−^ mice with spontaneous colitis has not yet been elucidated, thus we investigated the potential protective role of ESM on the effects of colitis and gut microbiota modulation in IL-10^−/−^ female mice.

## Materials and Methods

### Animal and Diets

The animal experiment was approved by the Animal Care and Use Committee of the University of Tokyo. Female wild-type (WT) mice and IL-10^−/−^ C57BL/6J mice, aged 9 or 10 weeks, bred in our laboratory were used in this study. The mice were individually housed in cages under a controlled temperature (23 ± 2°C), 12 h light-dark cycle environment (lights on from 08:00 a.m. to 20:00 p.m.) with a relative humidity of 40–60%. The mice were acclimatized for 1 week and then randomly divided into three groups based on their genetic types and diets: WT (WT mice; control diet of AIN93G, *n* = 10), KO (IL-10^−/−^ mice; control diet, *n* = 16), KOE (IL-10^−/−^ mice; 8% ESM diet, *n* = 16) for 19 weeks. The dietary composition is shown in Supplementary Table S1. As the digestibility of ESM is ~46%, the ESM diet was adjusted with cornstarch and casein to maintain the caloric and protein balance. Animals had free access to the experimental diet and tap water.

### Sample Collection and Cecal Short-Chain Fatty Acid Analysis

At the end of experimental period, all 12 h-fasted mice were anesthetized using isoflurane before sacrificing. After sacrificing the mice, the tissues and cecal contents were collected, weighed, and stored at −80°C prior to analysis. The concentration of short-chain fatty acids (SCFAs) in cecal contents was measured using ion-exclusion high-performance liquid chromatography (HPLC), as described in a previous study (18). Briefly, 50 mg cecal contents were mixed with 100 μl distilled water and 15 μl 12% (v/v) perchloric acid. After centrifuging the mixture for 10 min at 13,000 × g, the collected supernatants were filtered with a 0.45 μm membrane filter (Cosmonice Filter W; Nacalai Tesque, Kyoto, Japan). The filtered samples were injected into an SIL-30AC autosampler (Shimadzu, Kyoto, Japan) for quantitative analysis.

### Bacterial 16S rRNA Gene Sequencing

Bacterial genomic DNA from mouse cecal content was extracted with the QIAamp Stool Mini Kit (Qiagen, Hilden, Germany) according to the manufacturer's instructions. The V3–V4 of the 16S rRNA gene were amplified with the primer set, 341F: 5′-CCTACGGGNGGCWGCAG-3′; and 806R: 5′-GACTACHVGGGTATCTAATCC-3′, and then incorporated with Illumina adapters for subsequent sequencing. Thereafter, the libraries were conducted with a single Illumina MiSeq run (MiSeq Reagent Kit V3, 600 cycles; Illumina, San Diego, CA, USA) according to the manufacturer's instructions.

### Bioinformatic Analysis

The collected sequencing data was analyzed with QIIME (v. 1.8.0) software and the method is described in our previous study. First, fast length adjustment of short reads (FLASH) (v.1.2.11) was used to assemble the paired-end reads. Assembled reads with an average *Q*-value < 25 were filtered out using in-house script. The same number of filter-passed reads was randomly selected from each sample and used for further analysis. The selected reads were then processed using the QIIME pipeline. The high-quality sequences were clustered into operational taxonomic units (OTUs) at 97% sequence similarity, and OTUs were assigned with the Greengenes database (v.13.8). The microbial functionality profiles were predicted using Phylogenetic Investigation of Communities by Reconstruction of Unobserved States (PICRUSt, v.2.1.4) to generate the Kyoto Encyclopedia of Genes and Genomes (KEGG) pathway. The predicted metagenomic data were aligned to the KEGG database, and the differences between groups were compared using STAMP (Welch's *t*-test, two-sided).

### RNA Extraction and Real-Time Quantitative Polymerase Chain Reaction

The colonic mucosa was scraped off gently with a blunt-edged glass slide. Total RNA was extracted from colonic mucosa using TRIzol™ reagent (Ambion® Life Technologies™, Foster City, CA, USA) and RNA Isolation Kit (NucleoSpin RNA II; Macherey Nagel, Düren, German). cDNA was synthesized from extracted total RNA with the PrimeScript™ RT Master Mix (Takara Bio, Tokyo, Japan) according to the manufacturer's instructions. The real-time quantitative polymerase chain reaction (qPCR) was performed using the Thermal Cycler Dice Real Time System TP800 (Takara Bio Inc.). The primer sequences used in the study are described in Supplementary Table S2. The expression level of ribosomal protein lateral stalk subunit P1 (*Rplp1*) was used as the internal reference for normalization of the target gene mRNA expression.

### Statistical Analysis

Data are expressed as mean ± standard error (SE) or boxplots with median, minimum, and maximum values. The normality and equal variances of the data were performed by Shapiro-wilk and Hartley's test firstly. Then, statistical analysis was performed using one-way ANOVA and Tukey-Kramer test. The survival analysis was performed using a Log-rank (Mantel-Cox) test in GraphPad Prism 9 (San Diego, CA). *P* < 0.05 was considered statistically significant.

## Results

### ESM Administration Significantly Improved Survival Rate and Body Composition in IL10^−/−^ Mice

**Figure 1:**
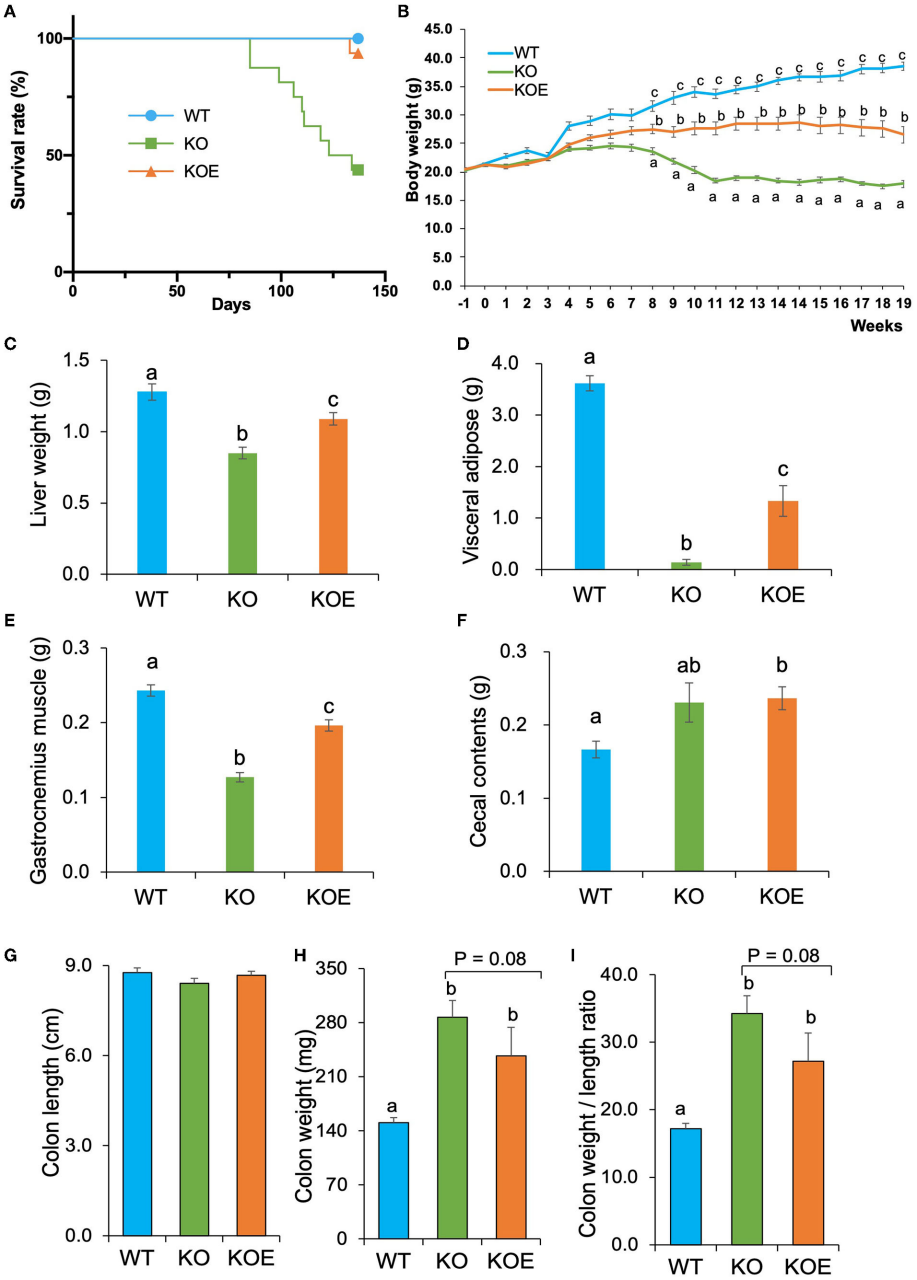
Effect of eggshell membrane (ESM) supplementation on the survival rate and body composition in IL10^−/−^ mice. **(A)** Survival rate in wild-type (WT) mice fed with control diet or IL10^−/−^ mice fed with control diet (KO group) or ESM supplemental diet (KOE group) for 19 weeks. During the treatment period, body weight **(B)** was measured for each group. After 19-week treatment, liver weight **(C)**, visceral fat pad weight **(D)**, gastrocnemius muscle weight **(E)**, cecal content weight **(F)**, colon length **(G)**, colon weight **(H)**, and colon weight to length ratio **(I)** were calculated (WT: *n* = 8, KO and KOE: *n* = 7, respectively). Data are presented as mean ± standard error. Superscript with different letters indicate significant difference at *P* < 0.05.

During the 19-week experimental period, there was no significant difference in the total food intake among the experimental groups. After the 19-week treatment, the survival rate was significantly lower in the KO group than in the WT group (*P* < 0.01); however, ESM supplementation significantly improved the survival rate of IL10^−/−^ mice (Figure 1A). From the 8- to 19-week treatment period, body weight was significantly lower in the KO group than in the WT group. ESM treatment largely reversed the body weight loss in IL10^−/−^ mice (Figure 1B). At necropsy, KO mice showed reduced liver, visceral fat, and gastrocnemius muscle weights, which was mitigated by ESM supplementation (Figures 1C–E). The cecal content weight in KOE group mice was higher than that in WT mice (Figure 1F). The mice colon length was not significantly different among the experimental groups (Figure 1G). However, the mice colon weight was significantly elevated in the KO group compared to the WT group (Figure 1H). Accordingly, the ratio of the colon weight (mg) to length (cm), an indirect indicator of edema and inflammation, increased in the KO group compared to the WT group, which was slightly alleviated by ESM supplementation (Figure 1I).

### ESM Administration Modulated the Composition of Gut Microbiota in IL10^−/−^ Mice

**Figure 2:**
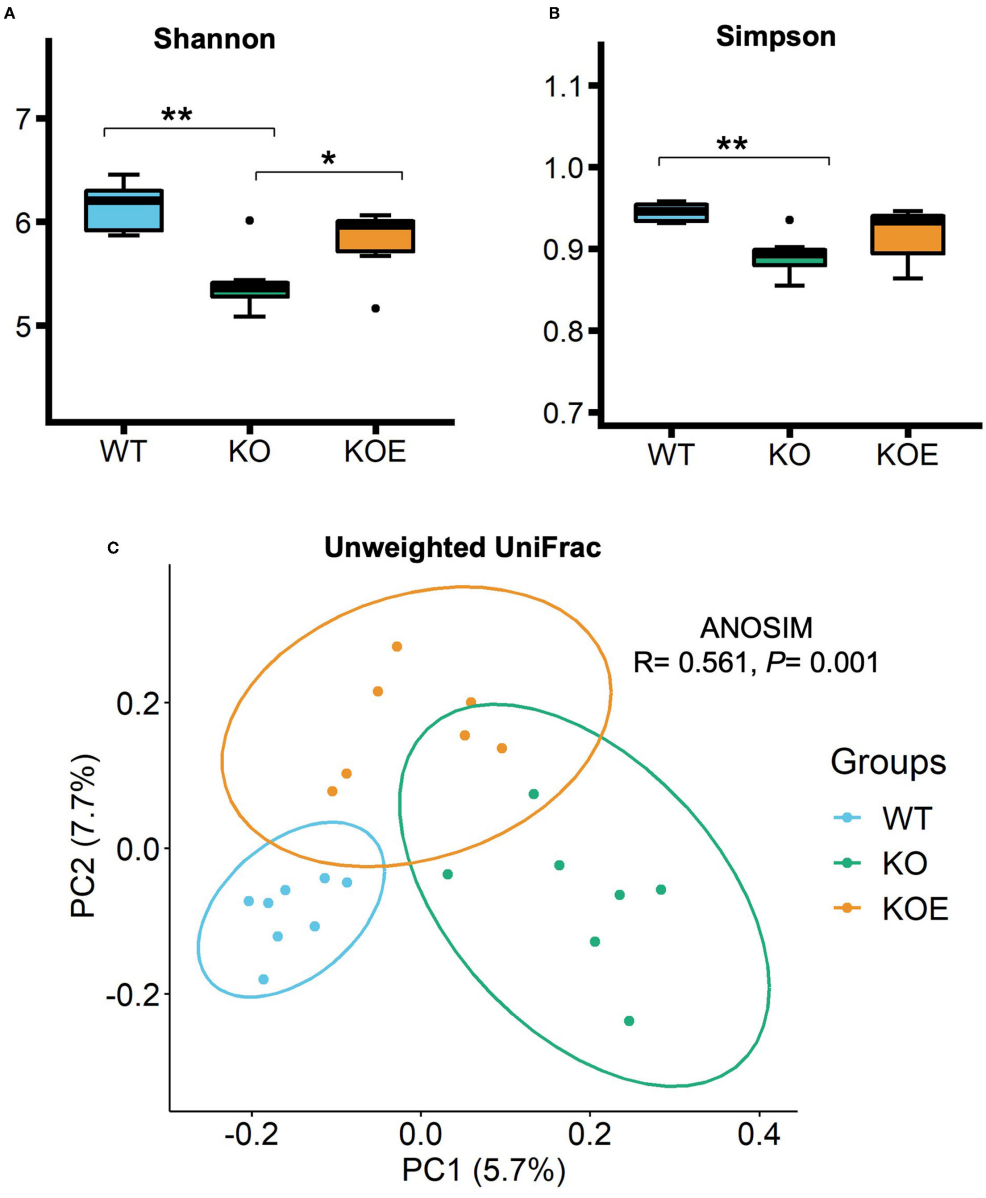
The α- and β-diversities of gut bacteria in mice of treatment groups [wild-type (WT): *n* = 8, IL10^−/−^ (KO) and KO with eggshell membrane supplementation (KOE): *n* = 7, respectively]. After the 19-week treatment, total bacterial DNA in cecal contents were extracted for 16S rRNA gene sequencing. Gut bacterial diversity and richness within samples were measured by **(A)** Shannon and **(B)** Simpson indices. The principal component analysis (PCoA) of Unweighted UniFrac **(C)** and ANOSIM analysis were conducted to compare the gut bacterial profiles among the three groups. Statistical analysis of α-diversity data was performed by ANOVA and the Tukey-Kramer test. ^*^P < 0.05, ^**^P < 0.01. The dots (•) in the boxplots indicate outliers.

To analyze the gut microbiota, a total of 28,313 filter-passed high-quality reads per sample were extracted for further QIIME pipeline analysis. Alpha diversity analysis showed that Shannon and Simpson indices were lower in the KO group than in the WT group (Figures 2A,B). However, Shannon index-based diversity was recovered by ESM supplementation. Principal component analysis of unweighted UniFrac analysis showed a distinct difference in the gut bacterial profiles between the WT and KO groups (Figure 2C). The KOE samples were clustered between the WT and KO groups.

**Figure 3:**
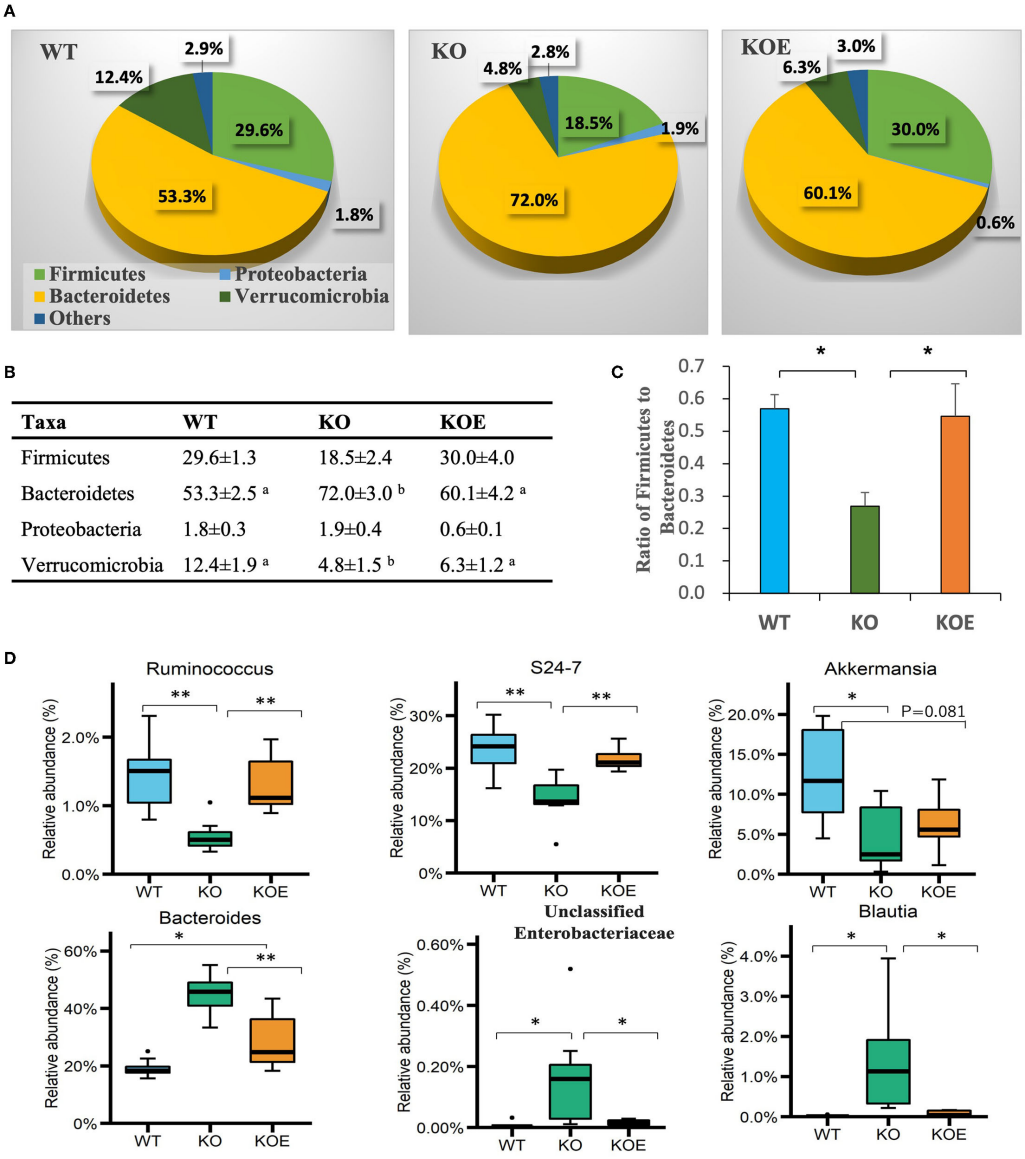
Effect of eggshell membrane (ESM) on gut bacterial composition in IL10^−/−^ (KO) mice [wild-type (WT): *n* = 8, KO and KO with ESM supplementation (KOE): *n* = 7, respectively]. Relative abundance of phyla is demonstrated in the pie graphs **(A)** and table **(B)**. **(C)** The ratio of Firmicutes to Bacteroidetes in the three groups. **(D)** The abundance of gut bacterial taxa at the genus level. Different superscript letters indicate significant differences at *P* < 0.05. ^*^P < 0.05, ^**^P < 0.01. The dots (•) in the boxplots indicate outliers.

Taxonomic analysis revealed that all treatment groups contained four dominant phyla: Firmicutes, Bacteroidetes, Proteobacteria, and Verrucomicrobia (Figures 3A,B). At the phylum level, the abundance of Bacteroidetes was enriched in KO mice and reversed in KOE mice (Figure 3B). The abundance of Verrucomicrobia was decreased in KO mice but recovered by ESM supplementation. The ratio of Firmicutes to Bacteroidetes was significantly lowered in the KO group compared to the WT group, which was almost completely restored by ESM treatment (Figure 3C). At the genus level, commensal bacteria, such as *Ruminococcus*, were significantly reduced in the KO group but recovered in KOE, compared to the WT group. The family Bacteroidales S24-7 also followed this trend (Figure 3D). In contrast, *Bacteroides*, unclassified Enterobacteriaceae, and *Blautia* were significantly enriched in the KO group over the WT and KOE groups (Figure 3D). Collectively, these data indicated that ESM treatment markedly modulated the composition of gut bacteria in IL10^−/−^ mice, resulting in a more similar bacterial structure to WT mice.

**Figure 4:**
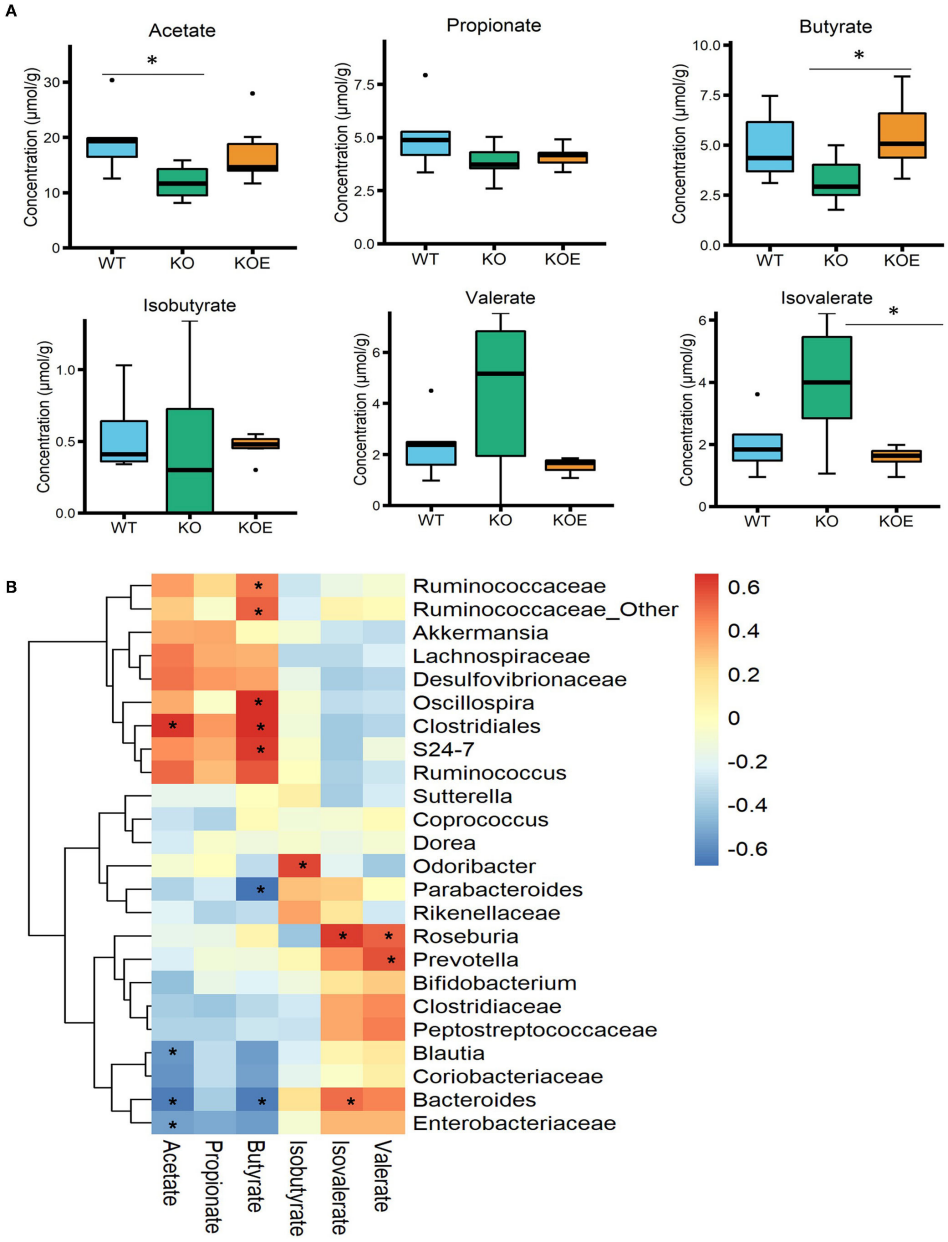
Effects of eggshell membrane (ESM) on the **(A)** levels of short-chain fatty acids (SCFAs) in cecal contents [wild-type (WT): *n* = 8, IL10^−/−^ (KO) and KO with ESM supplementation (KOE): *n* = 7, respectively]. **(B)** The heatmap showing the Pearson correlation between SCFAs and the relative abundance of the selected taxa at the family level. ^*^P < 0.05. The dots (•) in the boxplots indicate outliers.

The concentrations of SCFAs in mice cecal contents were quantified using a HPLC system. The level of butyrate, which is considered a beneficial bacterial metabolite in the intestines, was significantly increased in the KOE group over the KO group (Figure 4A). The levels of other SCFAs, including acetate, propionate, isobutyrate, and valerate, were not altered by ESM treatment (Figure 4A). The concentration of isovalerate was significantly lower in the KOE group than the KO group. To understand the relationship between specific bacterial taxa and cecal SCFAs, a Pearson-correlation heatmap was generated by selecting taxa at the family level and the levels of cecal SCFAs (Figure 4B). Particularly, a strong positive correlation was observed between Ruminococcaceae, Oscillospira, Clostridiales, and Bacteroidales S24-7 and the level of cecal butyrate, respectively. *Bacteroides* showed a positive correlation with isovalerate levels and a negative correlation with acetate and butyrate levels.

To obtain a better understanding of the influence of ESM supplementation on gut bacteria, PICRUSt analysis was conducted to predict the bacterial gene functional profiles based on the 16S rRNA gene sequences. There are 28 KEGG pathways associated with gut microbiota that were significantly altered in the KO group compared to the WT group (Supplementary Figure S1A). ESM supplementation affected 21 KEGG pathways when compared to the KO group. Among them, 14 pathways (marked with ^*^) that were altered in the KO group were recovered by ESM treatment (Supplementary Figure S1B).

### ESM Inhibited the Augmented Proinflammatory Gene Expression in IL10^−/−^ Mice

**Figure 5:**
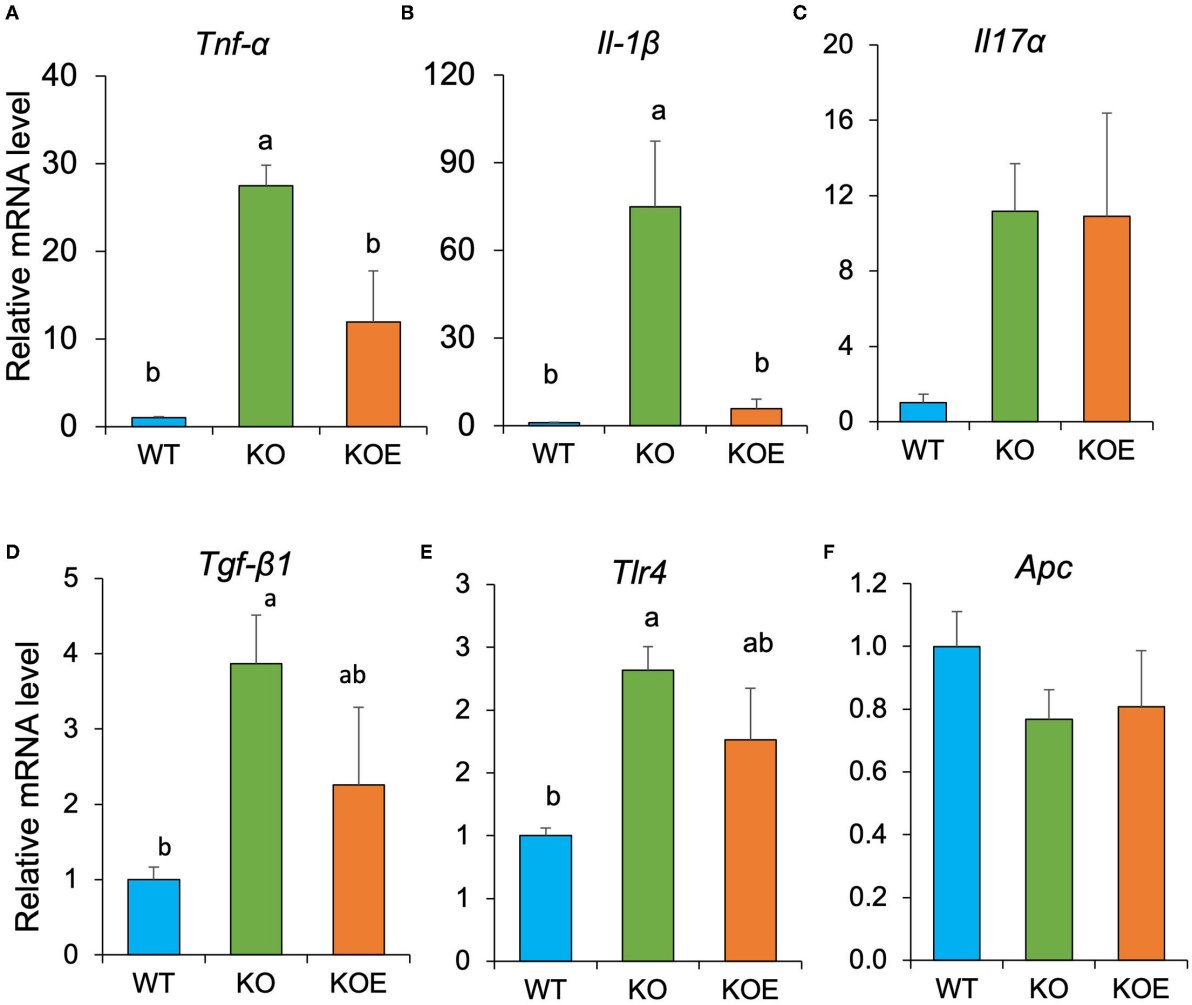
Eggshell membrane (ESM) modulates relative gene expression of several proinflammatory cytokines relative to RPLP1 in the mouse colonic mucosa. After the 19-week treatment, relative mRNA expression of *Tnf-*α **(A)**, *Il1*β **(B)**, *Il17a* **(C)**, *Tgf-*β*1* **(D)**, *Tlr4* **(E)**, and *Apc* **(F)** in colonic mucosa were examined using quantitative real-time polymerase chain reaction (qPCR). Data are presented as mean ± standard error. Statistical analysis was performed by one-way ANOVA and the Tukey-Kramer test. Superscript with different letters indicate significant difference at *P* < 0.05.

To further investigate the molecular mechanisms responsible for the observed beneficial effects of ESM on disease indices of IL10-KO mice, we analyzed relative gene expression in the mouse colonic mucosa with qPCR. The result showed the relative mRNA levels of *Tnf-*α and *Il1*β were dramatically upregulated in IL10^−/−^ mice, however, the gene expression was significantly inhibited by ESM treatment (Figure 5). The expression of the other genes related to intestinal homeostatic functions, including *Il17, Tgf-*β*1, Tlr4*, and *Apc*, were not significantly altered by ESM supplementation.

## Discussion

Eggshell membrane (ESM), primarily composed of collagen-like proteins, is considered as a safety and novel functional dietary ingredient. In this study, IL-10^−/−^ mice, as a spontaneous colitis model, were used to explore the effect of ESM on colitis. The survival rate and body weight loss were ameliorated following ESM treatment in the IL-10^−/−^ mice. Coincidently, the reduced tissue weights in the liver, visceral adipose, and gastrocnemius muscles were reversed following ESM treatment by the end of experimental period. These findings suggested that the colitis mouse model was successfully constructed, and ESM supplementation was effective in ameliorating some symptoms of colitis.

The alteration of gut bacterial composition is considered to play a critical role in the onset and progression of IBD. Therefore, we examined the effects of ESM on the composition of gut bacteria via 16S rRNA gene sequencing. Our results indicated that bacterial diversity was reduced in IL-10^−/−^ mice, but this was reversed by ESM supplementation. Moreover, the gut microbiota of the three groups was clustered into different groups, where the KOE group was positioned between the WT and KO groups. These results suggest that the gut dysbiosis observed in colitis mouse model might be improved by ESM treatment. Firmicutes and Bacteroidetes were dominant in the mice intestine according to the taxonomic analysis. The Firmicutes / Bacteroidetes (F/B) ratio was reduced in IL-10^−/−^ mice but was recovered to a normal level (similar to WT) following ESM treatment. The F/B ratio is generally considered to play an important role in maintaining regular gut homeostasis. The altered F/B ratio is a marker of dysbiosis, whereby an increase is normally observed with obesity, and a decrease is associated with IBD (19). However, an increased or unchanged F/B ratio was reported in other previous studies in IL-10^−/−^ mice (7, 20). This disparity may be attributed to the difference in housing conditions, maturity or other environmental factors.

At the genus level, *Ruminococcus* was significantly decreased in the KO group, but recovered in KOE mice, compared to the WT group. The family Bacteroidales S24-7 followed the same trend. The symbiont of the genus *Ruminococcus* often inhabits both the animal and human gastrointestinal tract, and can use fermentable carbohydrates for growth. For instance, *Ruminococcus bromii* has the ability to ferment resistant starch and subsequently contribute dramatically to butyrate production in the gut (21). Consistently, our results showed that treatment with ESM enhanced the production of butyrate, which is positively associated with the elevated level of Ruminococcaceae. Meanwhile, members of the family S24-7, recently renamed as Muribaculaceae, are dominant in the murine gut microbiota and have been identified in the gastrointestinal tract of other animals (22). The abundance of S24-7 has been shown to decrease in mice fed with a high-fat diet but to increase in those fed with dietary fibers (23, 24). In addition, the abundance of S24-7 recovered to a higher level following treatment-induced colitis remission in a mouse model (25). In our study, S24-7 was found to have a positive correlation with cecal butyrate. These results imply that S24-7 may play a critical role in the amelioration of colitis by treatment with ESM.

In contrast, *Bacteroides*, unclassified Enterobacteriaceae, and *Blautia* were significantly higher in the KO group than in WT or KOE groups. The family Enterobacteriaceae contains symbiotic bacteria and several well-known pathogens, such as *Escherichia coli, Klebsiella*, and *Shigella*. An increased abundance of Enterobacteriaceae has been reported in both IBD patients and mouse colitis models (26, 27). Consistently, our previous study showed that ESM treatment decreased the abundance of Enterobacteriaceae in DSS-induced IBD model mice (16). The proinflammatory properties of Enterobacteriaceae have been well studied, but the mechanism by which Enterobacteriaceae is enriched in IBD is not fully clear. Meanwhile, the commensal bacterium *Blautia* is a dominant genus in the gut microbiota that plays a critical role in maintaining gut environmental balance and preventing gut inflammation through regulation of intestinal regulatory T cells and production of SCFAs (28). Decreased levels of *Blautia* have been found in patients with Crohn's disease or colorectal cancer (29, 30). However, several studies found higher levels of *Blautia* in the feces of IBD patients and ulcerative colitis than in healthy individuals (31, 32). Given these conflicting results, it will be interesting to explore the role of *Blautia* in the etiology of colitis. Due to the current shortcomings of 16S rRNA gene sequencing for gut microbiota, this study could not identify the gut bacterial taxa at the species or strain levels. Therefore, more in-depth studies should be performed to investigate the detailed roles of the gut microbiota, especially at the species or even strain levels.

Through the analysis of relative gene expression in the mouse colonic mucosa with qPCR, we confirmed aggravated colon inflammation through the increased expression of proinflammatory gene mRNA, such as *Tnf-*α and *Il1*β. However, ESM supplementation ameliorated the colon inflammatory state by downregulating these proinflammatory genes. Previous studies have showed that butyrate exerts anti-inflammatory effects by activating GPR41 and GPR43 and inhibiting NFκB activation (33, 34). Together, these results indicated the possibility that ESM ameliorates the colon inflammatory state through the modulation of gut microbiota and its metabolites. Further study is required to confirm this possibility.

## Conclusions

In conclusion, the current study demonstrated the effectiveness of ESM supplementation in improving the survival rate and ameliorating the reduction of body weight, body fat, and muscle volume in IL10^−/−^ mice. Furthermore, ESM supplementation improved the imbalanced gut microbiota composition, as indicated by the higher bacterial diversity and richness, the increased abundance of commensal gut bacteria (*Ruminococcus* and Bacteroidales S24-7), and the decreased level of potentially harmful bacteria (Enterobacteriaceae) in IL10^−/−^ mice. Moreover, ESM supplementation promoted the production of butyrate in the gut and downregulated proinflammatory cytokine gene expression in the colon. These findings suggest that ESM could be used as a dietary intervention in colitis treatment.

## Data Availability Statement

The datasets presented in this study can be found in online repositories. The name of the repository and accession number can be found below: DDBJ; DRA013317.

## Ethics Statement

The animal study was reviewed and approved by the Animal Care and Use Committee of the University of Tokyo.

## Author Contributions

HJ, HK, and YH: conceptualization. HJ: methodology, supervision, and project administration. YY: formal analysis, writing original draft preparation, and visualization. WL, KF, and XL: data curation. HJ and HK: writing review and editing and funding acquisition. YH: resources. All authors have read and agreed to the published version of the manuscript.

## Funding

This research was funded in part by Grant-in-Aid, grant number 18K11095 from the Japan Society for the Promotion of Science (JSPS).

## Conflict of Interest

YH is working as a researcher in ALMADO Inc. in Tokyo. The remaining authors declare that the research was conducted in the absence of any commercial or financial relationships that could be construed as a potential conflict of interest.

## Publisher's Note

All claims expressed in this article are solely those of the authors and do not necessarily represent those of their affiliated organizations, or those of the publisher, the editors and the reviewers. Any product that may be evaluated in this article, or claim that may be made by its manufacturer, is not guaranteed or endorsed by the publisher.
