# Supplementary material for: Dietary Eggshell Membrane Powder Improves Survival Rate and Ameliorates Gut Dysbiosis in Interleukin-10 Knockout Mice
Source: Front Nutr. 2022 May 19;9:895665. doi: 10.3389/fnut.2022.895665 (PMC9162118; doi:10.3389/fnut.2022.895665)
Supplement: Supplementary file 1 [file Table_1.docx]

Supplementary Material

**Supplementary Table 1.** Composition of experimental diets

| Ingredient | WT/ KO (%, w/w) | KOE (%, w/w) |
| --- | --- | --- |
| ESM^1^ | - | 8.00 |
| L-Cysine | 0.30 | 0.30 |
| Casein^2^ | 20.00 | 16.30 |
| Vitamin mixture^3^ | 1.00 | 1.00 |
| Mineral mixture^3^ | 3.50 | 3.50 |
| Cellulose | 5.00 | 5.00 |
| Sucrose | 10.25 | 10.30 |
| Corn starch | 39.75 | 35.40 |

^1^ESM: Eggshell membrane powder.

^2^Casein: net protein content, 87% (w/w).

^3^American Institute for Nutrition (AIN-93).
