# Supplementary material for: Dietary Eggshell Membrane Powder Improves Survival Rate and Ameliorates Gut Dysbiosis in Interleukin-10 Knockout Mice
Source: Front Nutr. 2022 May 19;9:895665. doi: 10.3389/fnut.2022.895665 (PMC9162118; doi:10.3389/fnut.2022.895665)
Supplement: Supplementary file 2 [file Table_2.DOCX]

Supplementary Material

**Supplementary Table 2.** The list of primers used in the study

| **Gene** | **Full name** |  | **Primer sequence (5’-3’)** |
| --- | --- | --- | --- |
| *Tnf-α* | Tumor necrosis factor alpha | Forward | GGCAGGTCTACTTTGGAGTCATTGC |
|  |  | Reverse | ACATTCGAGGCTCCAGTGAATTCGG |
| *Il1β* | Interleukin 1 beta | Forward | TGGTGTGTGACGTTCCCATTAG |
|  |  | Reverse | GGTTGATATTCTGTCCATTGAGGTG |
| *IL17a* | Interleukin 17 alpha | Forward | ATCCCTCAAAGCTCAGCGTGTC |
|  |  | Reverse | GGGTCTTCATTGCGGTGGAGAG |
| *Tgf-β1* | Transforming growth factor beta 1 | Forward | CCTGTCCAAACTAAGGC |
|  |  | Reverse | GGTTTTCTCATAGATGGCG |
| *Tlr4* | Toll-like receptor 4 | Forward | CAAACTGGAACATAGCCACCT |
|  |  | Reverse | AGGAGTTTCTGTGAGAGGGAAG |
| *Apc* | Adenomatous polyposis coli | Forward | GGCTCGAAAATGGGGTCCAA |
|  |  | Reverse | AGCGTAGTTTCACTCCGGG |
| *Rplp1* | Ribosomal protein lateral stalk subunit P1 | Forward | ATCTACTCCGCCCTCATCCT |
|  |  | Reverse | CAGATGAGGCTCCCAATGTT |
