# Supplementary material for: Dietary Eggshell Membrane Powder Improves Survival Rate and Ameliorates Gut Dysbiosis in Interleukin-10 Knockout Mice
Source: Front Nutr. 2022 May 19;9:895665. doi: 10.3389/fnut.2022.895665 (PMC9162118; doi:10.3389/fnut.2022.895665)
Supplement: Supplementary file 4 [file Image_1.pdf]

Supplementary Material

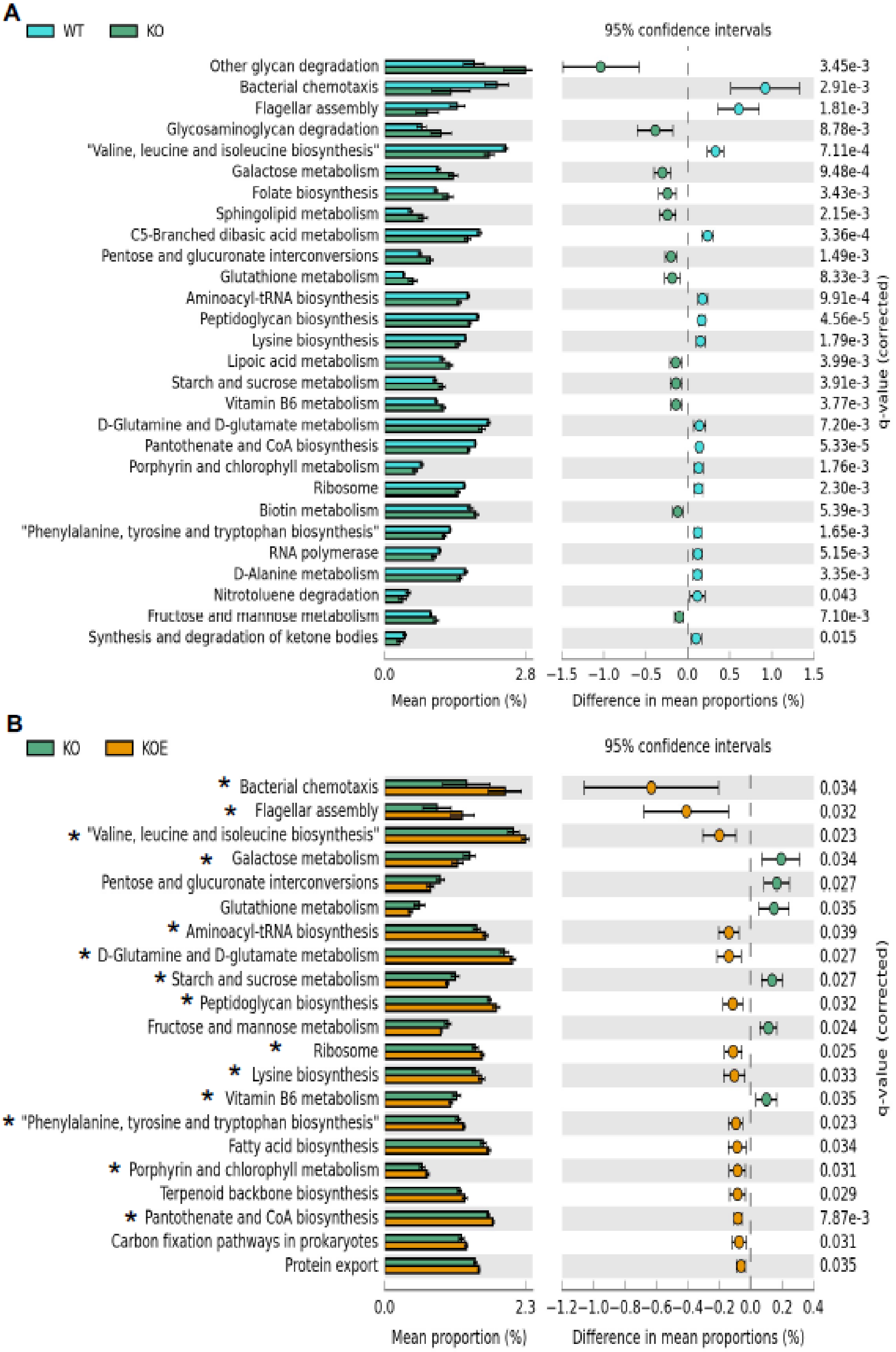

Supplementary Figure 1. Prediction of bacterial genes functions to make inferences from KEGG annotated databases using PICRUSt analysis (WT: n = 8, KO and KOE: n = 7, respectively). (A) Collectively, 28 KEGG pathways were statistically altered in the WT group compared to that in KO group. (B) In total, 14 KEGG pathways (marked with *) were recovered by ESM treatment. The mean proportion of each pathway is displayed in the bar graphs on the left.
